# Evaluation of Hydrogen Peroxide Fumigation and Heat Treatment for Standard Emergency Arthropod Inactivation in BSL-3 Insectaries

**DOI:** 10.3389/fbioe.2020.602937

**Published:** 2020-11-16

**Authors:** Irina Häcker, Roland Koller, Gerrit Eichner, Jakob Martin, Eleni Liapi, Johanna Rühl, Tanja Rehling, Marc F. Schetelig

**Affiliations:** ^1^Department of Insect Biotechnology in Plant Protection, Institute for Insect Biotechnology, Justus-Liebig-University Gießen, Gießen, Germany; ^2^Department of Insect Pest and Vector Control, Division of Bioresources, Fraunhofer Institute for Molecular Biology and Applied Ecology (IME), Gießen, Germany; ^3^Ortner Reinraumtechnik GmbH (Ortner Cleanrooms Unlimited), Villach, Austria; ^4^Mathematical Institute, Justus-Liebig-University Gießen, Gießen, Germany; ^5^Department of Biochemistry and Biotechnology, University of Thessaly, Larissa, Greece

**Keywords:** BSL-3 insectary, vector insects, infectious disease, gene drive, containment, hydrogen peroxide, heat susceptibility, inactivation procedures

## Abstract

Climate change and global movements of people and goods have accelerated the spread of invasive species, including insects that vector infectious diseases, which threaten the health of more than half of the world’s population. Increasing research efforts to control these diseases include the study of vector – pathogen interactions, involving the handling of pathogen-infected vector insects under biosafety level (BSL) 2 and 3 conditions. Like microbiology BSL-3 laboratories, BSL-3 insectaries are usually subjected to fixed-term or emergency room decontamination using recognized methods such as hydrogen peroxide (H_2_O_2_) or formaldehyde fumigation. While these procedures have been standardized and approved for the inactivation of diverse pathogens on surfaces, to date, there are no current standards for effective room-wide inactivation of insects in BSL-3 facilities in case of an emergency such as the accidental release of a large number of infected vectors. As H_2_O_2_ is often used for standard room decontamination in BSL-3 facilities, we evaluated H_2_O_2_ fumigation as a potential standard method for the safe, room-wide deactivation of insects in BSL-3 insectaries in comparison to heat treatment. To account for physiological diversity in vector insect species, six species from three different orders were tested. For the H_2_O_2_ fumigation we observed a strong but also varying resilience across all species. Lethal exposure time for the tested dipterans ranged from nine to more than 24 h. Furthermore, the coleopteran, *Tribolium castaneum*, did not respond to continuous H_2_O_2_ exposure for 48 h under standard room decontamination conditions. In contrast, temperatures of 50°C effectively killed all the tested species within 2 to 10 min. The response to lower temperatures (40–48°C) again showed a strong variation between species. In summary, results suggest that H_2_O_2_ fumigation, especially in cases where a gas generator is part of the laboratory equipment, may be used for the inactivation of selected species but is not suitable as a general emergency insect inactivation method under normal room decontamination conditions. In contrast, heat treatment at 48 to 50°C has the potential to be developed as an approved standard procedure for the effective inactivation of insects in BSL-3 facilities.

## Introduction

The increasing worldwide threat by emerging and re-emerging vector-borne infectious diseases has boosted related research activities to study vector-pathogen interactions and enable the development of diagnostic tests, vaccines, and drugs. For example, fewer than 40 Zika virus publications per year were registered in the PubMed database between 1977 and 2015, but more than 1500 per year from 2016–2019 (based on the PubMed search query “Zika virus”). Research involving vector-borne diseases often ultimately involves experiments on pathogen-infected insects, requiring specific containment and decontamination measures, which depend on the biosafety classification of the pathogen. There are four globally recognized biosafety levels (BSL) defined by the Centers for Disease Control and Prevention in the United States that have been adopted in Europe under Directive 90/679/EEC (Council of the European Union, 1990). The same designations are known as containment levels in Canada. The lowest biosafety level, BSL-1, is appropriate when there is little danger to personnel or the environment. In contrast, the highest level (BSL-4) is required for easily transmitted pathogens that cause severe to fatal human diseases for which there are no available vaccines or treatments.

Most well-known arthropod-borne diseases are classified as BSL-2 or BSL-3, including dengue, chikungunya fever, Zika fever, West Nile fever, Yellow fever, and malaria. In addition, gene drive systems are being developed worldwide to suppress vector populations or replace them with disease-refractory insects. Such projects have recently been classified as BSL-3 in Germany, for example, and therefore also require BSL-3 insectaries. General mandatory BSL-2 and BSL-3 precautions cover aspects such as special protective personal clothing and equipment, approved disinfectants, and surface and room decontamination procedures. However, these regulations were developed for microbiology laboratories and animal houses. While there are guidelines for arthropod containment laboratories and safe working procedures (Scott, 2005; Tabachnick, 2006; American Committee Of Medical Entomology, 2019), to our knowledge, there are no legal regulations for BSL-2 or BSL-3 insectaries. Accordingly, the microbiology safety precautions have been adapted individually by BSL-3 insectary users, in cooperation with local authorities, to account for the specific challenges of working with infected arthropods, i.e., small and highly mobile (flying) pathogen carriers (personal communication). This includes best-practice solutions for the containment of insects such as air curtains, airlocks, or both. For the inactivation of escapees, the only available solution so far seems to be the use of contact insecticides, not only for the inactivation of individual escapees but also for worst-case scenarios like the accidental release of many insects. However, given the expectable increasing number of research projects involving insect-borne diseases, an effective, standardized, and widely accepted room-wide insect inactivation procedure for BSL-2 and BSL-3 insectaries would be important.

Fumigation procedures have been developed for the routine room-wide decontamination of high-level biosafety facilities such as BSL-3 and BSL-4 microbiology containment laboratories, animal houses, and hospital environments. They are accepted as safe and validated disinfection methods. Formamide was first used for microbiological decontamination, starting in the early 20th century (Dreyfus, 1914). It inactivates viruses, microbial cells, and even resistant microbial spores (Rogers et al., 2007; Gordon et al., 2012). It is also recognized as a disinfectant by the Robert Koch Institute (Robert Koch Institut, 2017). The vapor is highly effective because it expands to infiltrate also small crevices. However, this leads to increased room pressure and, therefore, to more stringent requirements for the room’s gas impermeability. Consequently, surrounding rooms have to be closed during the procedure. Furthermore, formamide forms toxic or irritant residues on surfaces, which subsequently need to be cleaned (Cheney and Collins, 1995; Krishnan et al., 2006; Gordon et al., 2012; Kaspari et al., 2014). Finally, since 2014, formamide is classified as a Class 1B carcinogen and Class 2 mutagen in Europe and has also been recognized as a human carcinogen in the United States (U.S Department of Health and Human Services, 2016). These drawbacks of formamide use led to the search for effective alternatives such as hydrogen peroxide (H_2_O_2_). H_2_O_2_ breaks down naturally into water and oxygen, thus not leaving toxic traces or requiring subsequent cleaning measures. Consequently, it has become more widely used than formamide for room-wide decontaminations in recent years. It has proven to be effective against a wide range of bacteria, spores, and viruses in various facility settings (Heckert et al., 1997; Krishnan et al., 2006; Pottage et al., 2010; Bentley et al., 2012; Goyal et al., 2014; Kaspari et al., 2014; Lemmen et al., 2015; Petit et al., 2017). Depending on the environmental conditions, H_2_O_2_ can react as a weak acid, a potent oxidizing, or a reducing agent. Moreover, it can easily form hydroxyl free radicals, a quality that is utilized for microbiological disinfection/decontamination. It is also used in some BSL-3 insectaries for the mandatory annual microbiological room decontamination (personal communication) in Germany. Thus, an H_2_O_2_ gas generator is often standard equipment in a BSL-3 insectary. H_2_O_2_ fumigation would therefore be a good candidate for the development as a recognized standard procedure for the safe, room-wide deactivation of insects in BSL-3 insectaries if its effectiveness against insects of diverse physiologies (as they occur in different vector insects like mosquitoes, sandflies, triatomine bugs, or ticks), could be established.

Alternatively, heat treatments can be used to inactivate insects. Already in the early 1900s, heat was extensively evaluated and applied to eliminate different stored-product insect pests and mites, predominantly in mills, like the red flour beetle *Tribolium castaneum*, the confused flour beetle *T. confusum*, the Mediterranean flour moth *Ephestia kuehniella*, and many others (Fields, 1992; Dean, 1993; Dowdy, 1999). While heat treatment for stored product pest control disappeared for some time, it has been revived and optimized in recent years as it offers an environmentally friendly alternative to chemical control (Hansen et al., 2011; Subramanyam et al., 2011; Porto et al., 2015; Valenti et al., 2018). Heat was also more effective for eliminating such pests in mills or other building structures like food processing facilities than fumigation. Provided that the heating time was sufficient to allow also low heat capacity materials to reach the target temperature, the heat also reached small crevices were the insects typically survived the fumigation procedures because they were less exposed to the gas (Dean, 1993). Similar circumstances could apply to insectaries, where insects might equally find small crevices hardly penetrated by the gas, especially when using H_2_O_2_, which in contrast to formamide, has no expansion properties. For the efficient inactivation of insects, we compared the effectiveness of fumigation to heating using practicable and standard H_2_O_2_ room decontamination conditions and simulating room temperatures of up to 50°C.

## Materials and Methods

### Insect Species

We tested six insect species representing three orders: (1) *Aedes aegypti* (Orlando strain, adults, mixed-sex; strain sourced from the Insect Transformation Facility, University of Maryland, United States), *Anopheles stephensi* (SD500 strain, adults, mixed-sex; strain provided by Dr. Andrew M. Blackborough, Imperial College, London, United Kingdom), *Drosophila melanogaster* [Oregon-R strain (Lindsley and Grell, 1968), adults, mixed-sex], and *Ceratitis capitata* (for H2O2 treatment: Vienna 8 strain, adults, males only; for heat treatment: *Egypt-II* strain, adults, mixed-sex; both strains sourced from the Insect Pest Control Laboratory, Seibersdorf, Austria) representing the Diptera; (2) *Tribolium castaneum* (San Bernardino laboratory strain, adults, mixed-sex; strain sourced from Fraunhofer IME-Bioresources) representing the Coleoptera; and (3) *Spilostethus pandurus* (wild collection, larvae and adults, mixed-sex; sampled in Portugal) representing the Hemiptera.

### Experimental Setup for the H_2_O_2_ Fumigation

**FIGURE 1:**
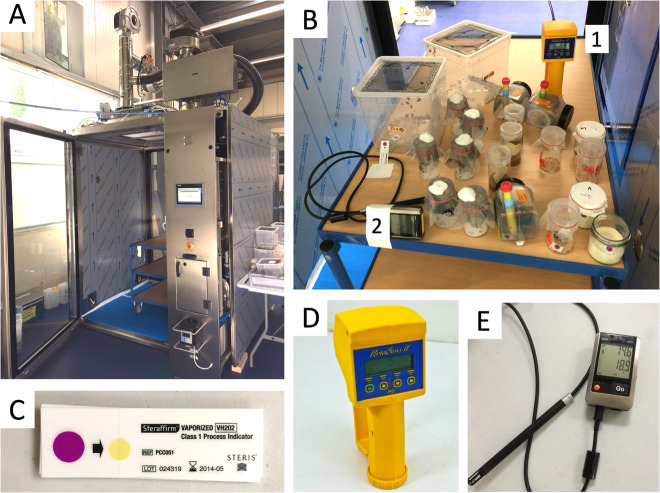
Equipment used for the H_2_O_2_ fumigation experiments at Ortner Reinraum GmbH. **(A)** airlock; **(B)** table within the airlock showing the setup of experimental cages and instruments [H_2_O_2_ gas sensor (1), see also **(D)** and data logger for temperature and humidity (2), see also **(E)**]; **(C)** Steris Steraffirm chemical H_2_O_2_ indicator strips; **(D)** ATI Portasens II portable gas detector used inside the airlock; **(E)** Testo 176H1 data logger for temperature and humidity monitoring inside and outside (control cohorts) of the airlock.

The experiments were conducted at the Ortner Reinraum GmbH testing site using a specially equipped H_2_O_2_ decontamination airlock (Figure 1A). H_2_O_2_ gas was generated using an ISU 2.0 system (Ortner Reinraum) from a 35% (v/v) H_2_O_2_ solution (Clamarin). The liquid H_2_O_2_ was vaporized in a generator-evaporator system to 125–135°C and mixed with an airstream preheated to 35°C flowing at ∼100 m^3^ h^–1^. The mixture was passed through integrated fans, filters, catalysts, and injection nozzles to achieve a highly turbulent air injection into the chamber. Decontamination was carried out as a dry process in a closed-loop system to prevent H_2_O_2_ condensation. In all experiments, the H_2_O_2_ concentration was slowly increased to the final concentration (400 or 1000 ppm) over 30 min to simulate a typical room decontamination cycle. Experimental insect cages were placed on a table within the airlock (Figure 1B). The control cohorts were kept on a table in the production hall next to the airlock. Reserve insects not used for the experiments were kept in metal transport boxes within the production hall. The food and water supply of the insects were checked daily. The penetration of H_2_O_2_ into the experimental vials and cages was monitored using Steraffirm Vaporized VH2O2 Class I Process Indicators (STERIS Life Sciences, Figure 1C). H_2_O_2_ concentration was measured and recorded by the airlock’s control unit (Supplementary Figure 1) and monitored using a PortaSens II gas detector (ATI, Figure 1D). Temperature and humidity inside and outside of the airlock were monitored using Testo 176H1 data loggers (Figure 1E). The temperature inside the airlock varied between 25 and 28°C during the experiments, and the relative humidity (RH) ranged from 50 to 100% (saturation). Depending on the outside weather conditions, the temperature next to the control cohorts in the production hall was between 20 and 25°C, the RH was 40–70%.

The airlock was available for a testing period of 5 days. Due to these time constraints and the required long incubation periods, each experiment could be conducted only once. Moreover, all long-term exposure experiments at 400 ppm H_2_O_2_ featured an interruption lasting ∼9 h during which the 1000 ppm experiments were completed and several interruptions lasting ∼1 h to assess the survival of the insects (Supplementary Figure 1).

### H_2_O_2_ Exposure Conditions

For all performed H_2_O_2_ exposures, the exact number of replicates and number of individuals per replicate are provided in Supplementary Table 1 (the 0 h values). Moreover, the table includes information about the type of housing of the insects during the exposure (*V* = 175 ml *Drosophila* vial covered with mesh, or “cage” = 20 cm × 20 cm × 20 cm cage with netting).

#### Mosquitoes

*Aedes aegypti* and *Anopheles stephensi* adults were placed in 175 ml *Drosophila* rearing vials (25–40 insects per vial) closed with a fine mesh, or housed in groups of 46 to 90 insects in 20 cm × 20 cm × 20 cm cages containing netting on opposite sides and in the lid for good aeration, and a fabric sleeve on one side for safe access to the cage (Supplementary Figures 2A–C). The insects were fed on 10% sucrose via soaked cotton pads accessible through the containers’ netting. Mosquitoes were 1 week old at the beginning of the experiments. The insects in vials were exposed to 400 ppm H_2_O_2_ for 7, 14, or 21 h, or to 1000 ppm H_2_O_2_ for 5 h. To determine the lethal exposure time LT_100_ at 400 ppm (all individuals dead), three vials and one cage were initially exposed for 7 h (*Aedes*) or 14 h (*Anopheles*). Exposure was then extended in 1 h intervals until the last individual was dead. Death was confirmed when insects no longer responded to tapping the closed vials or stimulation with a brush.

The effect of H_2_O_2_ on *Aedes* eggs was tested with a 3 months old egg collection stored under standard rearing conditions in a zipper bag wrapped in a humid paper towel. For the H_2_O_2_ exposure (400 ppm H_2_O_2_ for 7, 14, or 21 h, and 1000 ppm H_2_O_2_ for 5 h) the paper with eggs was placed openly on the table inside the airlock. After H_2_O_2_ exposure, the eggs were visually inspected, re-humidified by spraying with water, and stored in a zipper bag. An unexposed control batch was re-humidified and stored separately. All eggs were counted and hatched approximately 1 week after H_2_O_2_ exposure by submerging the eggs in degassed water supplemented with a few crumbs of TabiMin fish food (Tetra) to synchronize hatching. Hatched larvae, eggs, and oviposition papers were transferred to rearing trays the next day and larvae were reared on TabiMin fish food until pupation. Pupae were collected and counted daily to determine the pupation rate of the treated and control eggs.

#### *Ceratitis capitata* (Medfly)

Flies were housed in standard 150-ml rearing cages with netting on opposite sides (35 insects per cage) and were supplied with water and a standard diet of 3:1 (v/v) sugar and yeast extract (Supplementary Figure 2D). Flies were 1 week old at the beginning of the experiments. H_2_O_2_ exposures were performed in standard rearing cages or in 20 cm × 20 cm × 20 cm cages as used for the mosquitoes, with a paper-lined floor. The flies were supplied with a cup of food and a wet cotton pad inside the cage during and after the experiments (Supplementary Figure 2E). Flies were exposed to 400 ppm H_2_O_2_ for a total of 26 h and to 1000 ppm for 5 h, with interruptions for lethality assessment after 14 and 21 h (400 ppm) or 3 and 4 h (1000 ppm). Survival was also monitored for 5 days (400 ppm) and 6 days (1000 ppm) after conclusion of the H_2_O_2_ treatment.

#### Drosophila melanogaster

Flies were housed in 175 ml *Drosophila* rearing vials covered with mesh (∼30 insects per vial) containing standard *Drosophila* diet [0.8% (w/v) agar, 1% (w/v) soy flour, 8% (w/v) corn flour, 1.8% (w/v) brewer’s yeast, 8% (w/v) malt, 2.2% (w/v) molasses, 0.2% (w/v) Nipagin, and 0.625% (v/v) Propionic acid] (Supplementary Figure 2F). Flies were 3–6 days old at the beginning of the experiments. Three vials were exposed to 400 ppm H_2_O_2_ for 47 h with lethality assessments after 7, 21, 35, and 42 h. Another two vials were exposed for 40 h and were checked after 14, 28, and 35 h. Two further vials were exposed to 1000 ppm H_2_O_2_ for 5 h with lethality assessments after 3 and 4 h. The development of larvae was monitored for 27 days after treatment. All the above experiments were conducted in the presence of food. In addition, one 400 ppm 9 h exposure was conducted with two vials of flies in the absence of food (Supplementary Figure 2A).

#### *Spilostethus pandurus* (Milkweed Bug)

Milkweed bugs were reared on organic sunflower seeds in 20 cm × 20 cm × 20 cm plastic cages with netting in the lid for aeration (Supplementary Figure 2G), each cage containing 25–27 *Sp. pandurus* larvae at different larval stages. A few larvae reached the adult stage during the experiments or post-treatment monitoring. Cages were sprayed with water twice a day to ensure sufficient humidity. The larvae were exposed to 400 ppm H_2_O_2_ for 47 h, or 1000 ppm H_2_O_2_ for 5 h, as described above for *D. melanogaster*. Surviving insects were monitored after the experiment for 5 days.

#### *Tribolium castaneum* (Red Flour Beetle)

Adult beetles were maintained on organic wheat flour in 100-ml glass vials covered with paper cloth (∼35 animals per vial). The experimental and control cohorts were transferred to 175 ml *Drosophila* rearing vials covered with mesh and were provided with a small amount of flour and some sawdust (Supplementary Figure 2H). Beetles were exposed to 400 ppm H_2_O_2_ for 47 h, or 1000 ppm H_2_O_2_ for 5 h, as described above for *D. melanogaster*. Surviving beetles were monitored after the experiment for 5 days.

### Insect Heat Treatments

To investigate the influence of temperature on insect survival, insects were individually placed into closed 1.5-ml reaction tubes and incubated in a heat block at different temperatures for varying time intervals. For *D. melanogaster* and *C. capitata*, cotton wool was placed in the upper part of the reaction tubes to prevent the insects from evading the heat treatment by moving into the tube’s lid. Each combination of temperature and time was assessed in 2–4 biological replicates over 9 months (i.e., insects of different replicates originated from different generations), with each replicate consisting of 20 individuals. After each incubation interval, knock-down of the insects was assessed immediately by tapping the tube and/or stimulation with a brush. All insects were subjected to a 24 h post-treatment monitoring (PTM) to verify the death, as in many cases, the observed post-incubation knock-down was not caused by death but by heat-induced temporary unconsciousness, and the insects recovered from the treatment within the following 24 h. Incubation times were elongated gradually for each temperature until no recovery was observed within 24 h post-treatment.

Insects incubated individually in reaction tubes at 25°C for the duration of the heat treatment plus the 24 h PTM served as non-treatment controls. If multiple temperatures or time intervals were tested on the same day with insects from the same cage, a single control cohort was used for all tested conditions. For *C. capitata, D. melanogaster*, and *T. castaneum*, insects of mixed sex were used for all the tests. As female *Aedes* mosquitoes in the daily rearing procedures are more resilient to stress such as shortage of water or changes in temperature than males (long-term personal observations) and also have a markedly longer life span (Häcker et al., 2017), only females were used for these tests. The age of insects used for the temperature tests was about 1 week for medfly and 3–10 days for *Drosophila*. For *Aedes*, different age groups of females between 1 and 29 days were assessed (see specifications in Supplementary Table 2). *T. castaneum* specimens were of mixed age (from a few days up to several weeks).

### Data Evaluation and Statistics

#### H_2_O_2_ Treatment

For statistical analyses and graphics of H_2_O_2_ treatments, R version 3.6.3 (R Core Team, 2020) together with the following add-on packages was used: readxl (version 1.3.1) (Wickham and Bryan, 2019) to import data from Excel files, survival (version 3.1–11) (Therneau, 2020) for the Kaplan-Meier survival curves and their graphs, and Exact (version 2.0) (Calhoun, 2019) for the exact comparisons of two binomial probabilities.

(a) Samples from different biological replicates of treatments (v) and controls (c) were pooled. Since the observed survival times were partially right-censored, the effect of H_2_O_2_ treatments over time (treatment period plus post-treatment monitoring) was analyzed using the non-parametric Kaplan-Meier estimator together with the log-rank test to compare the survival curves of different treatments. The significance level was set to 0.05.

(b) Selective comparisons of proportions of survivors between treatment and control at specific early time points (i.e., the end of H_2_O_2_ treatment in cases of post-treatment monitoring) was performed using Boschloo’s exact test (two-sided) of equal proportions for two independent random samples, because there were no censored survival times at those early time points.

#### Heat Treatment

The highest number of alive insects in each treatment cohort recorded between treatment termination and the end of the 24 h PTM was used for the analysis of survival numbers. The number of alive insects in the 25°C control at the respective timepoint was used as corresponding no-treatment survival number. Box and whisker charts of the survival numbers were created using the box and whisker chart function of MS Excel, selecting the inclusive median and displaying outliers and the mean.

## Results

### A 1 h Exposure at 300 ppm H_2_O_2_ Does Not Affect Insect Fitness

Hydrogen peroxide room decontamination is typically performed at 300–500 ppm H_2_O_2_. To our knowledge, the sensitivity of insects to H_2_O_2_ fumigation has not yet been investigated. For a first assessment, an initial 1 h test run at 300 ppm with one vial each of *T. castaneum* and *Ae. aegypti* was performed. Initial H_2_O_2_ influx into the airlock caused the mosquitoes to start flying and mating. After ∼20 min, female mosquitoes were observed sitting on the bottom of the vial, cleaning their proboscis with their forelegs. The beetles showed no changed behavior. After 1 h, the airlock was aerated, and the vials were removed. At this point, the mosquitoes started flying again and both insect species resumed normal, lively behavior, showing that the 1 h fumigation had no effect.

### Seven to 21 h Exposures Affect Only Mosquito Viability

Given the initial test results, the H_2_O_2_ dose was increased to 400 ppm and the exposure time to 7 h. In addition to *Ae. aegypti* and *T. castaneum* (three vials each), now also the other species were included [three standard rearing cages of *C. capitata* (Supplementary Figure 2D), two cages of *Sp. pandurus* (Supplementary Figure 2G), and three vials each of *An. stephensi* (Supplementary Figure 2C) and *D. melanogaster* (Supplementary Figure 2F)]. Chemical H_2_O_2_ indicators were placed in one *Sp. pandurus* and one *C. capitata* cage, in one vial of *T. castaneum*, and in two flour containers from which the beetles had been removed. One indicator strip was placed directly on the table (Figure 1B) to compare the speed of color change within the containers and in the free air space. Moreover, the control cages for each species were set up next to the airlock to monitor the insects’ survival in the absence of H_2_O_2_. These cages were also used as controls for all subsequent experiments.

**FIGURE 2:**
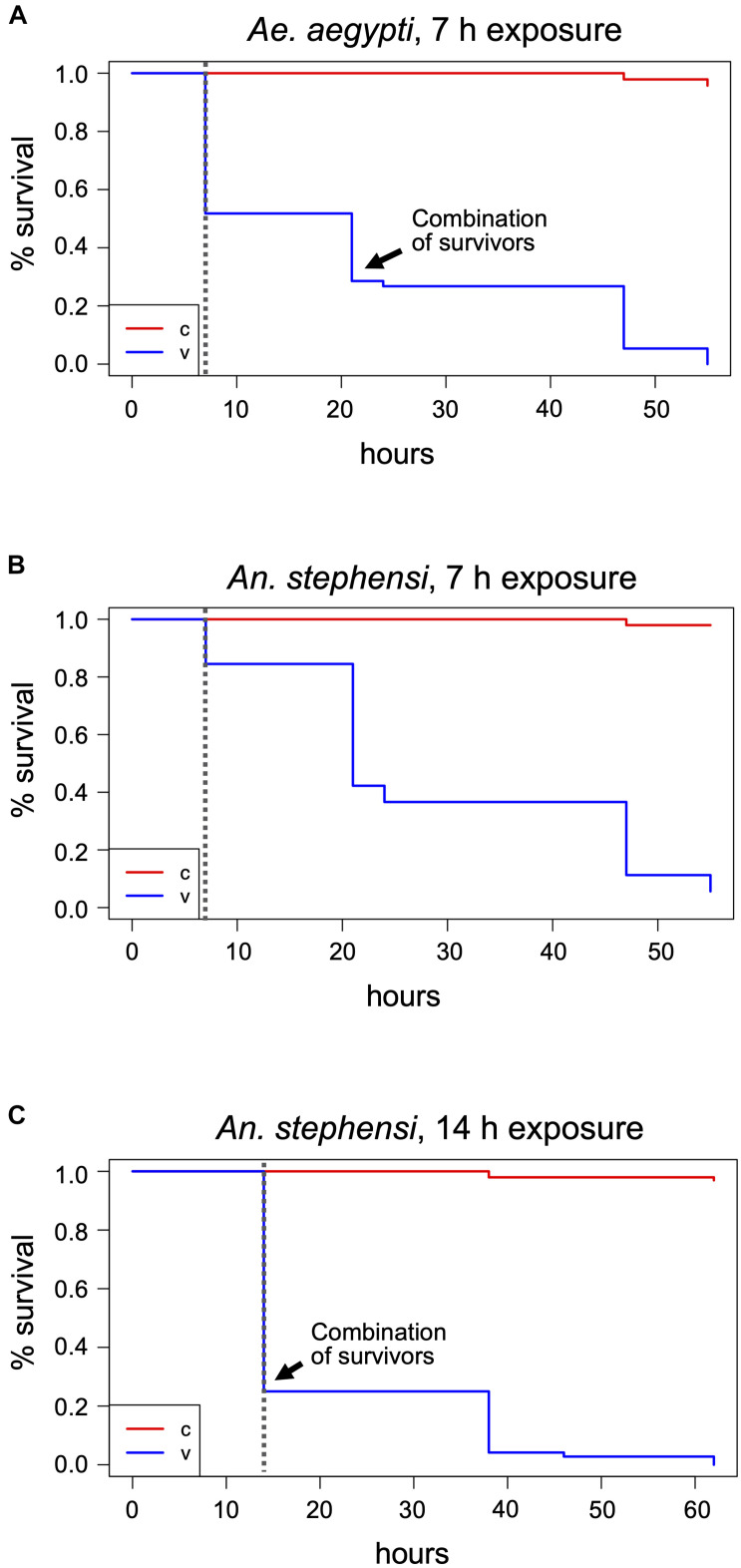
Mosquito treatments at 400 ppm H_2_O_2_ and post-treatment survival monitoring. Adult mosquitoes were subjected to 400 ppm H_2_O_2_ treatments for 7 h **(A,B)** or 14 h **(C)** and dead individuals counted after exposure termination. Survivors were further monitored until the time of death. The Kaplan-Meier estimator was used to estimate survival. Survival numbers from the two biological replicates per species were combined for the analysis. The curves show the probability of survival per time interval. The dashed vertical line indicates the end of H_2_O_2_ exposure. The black arrow indicates the time point where the remaining survivors of the two vials were combined into one for further monitoring, v, treatment cohort; c, control cohort.

During the 30 min visual monitoring of the indicators before condensation on the airlock’s glass door obscured the view, it became apparent that the insect containers markedly delayed the increase in H_2_O_2_ concentration compared to the free air space. After 7 h, the indicators in all containers had turned to yellow, proving the gas’s prolonged presence. Visible effects of the treatment could be seen with the mosquitoes and medflies. About half of the *Aedes* mosquitoes (predominantly males) were dead (Figure 2A and Supplementary Table 1), while the rest showed weak movement. *An. stephensi* was more resistant to the treatment, with only about 20% deaths (again predominantly males). Here, death could only be assessed roughly as many individuals were still flying or attempting to fly, preventing the opening of the vials and individual assessment of death (Figure 2B and Supplementary Table 1). Two of the three vials (V1 and V2) of each mosquito species were then placed next to the controls for the longer-term evaluation of treatment effects, while the third vial (V3) was returned for extended exposure (see below). In the treated vials placed with the controls, none of the individuals showed improvement of the health condition, and all *Aedes* died within 48 h post-treatment, while after 48 h, four *Anopheles* females still remained alive (Figures 2A,B and Supplementary Table 1). During the treatment and PTM period, only 4 (*Aedes*) and 3 (*Anopheles*) of the more than 90 control specimens each died (*p* < 2^–16^, Chi-square test).

In medfly containers, all flies were alive and lively after 7 h but had fallen onto their backs during the exposure and their wings had stuck to the plastic bottom of the rearing container due to the humidity. They were able to move their head and legs, and when freed, they walked but could not fly. The other three species (*D. melanogaster, Sp. pandurus*, and *T. castaneum*) showed no visible effects of the 7 h H_2_O_2_ exposure.

One of each mosquito vial, and all *D. melanogaster, Sp. pandurus*, and *T. castaneum* containers were returned to the airlock for 14 h. Additionally, fresh containers of each species were added to simultaneously assess the effects of 14 and 21 h H_2_O_2_ exposures (Supplementary Figure 1). At the end of these extended treatments, all *Aedes* mosquitoes were dead (Supplementary Table 1), whereas 25–30% of the *Anopheles* (predominantly females) survived exposure for 14 h but were unable to fly and died within the next 48 h (Figure 2C and Supplementary Table 1). None of the *Anopheles* survived exposure to H_2_O_2_ for 21 h. All mosquito specimens from these tests had a yellowish, bleached appearance.

Fourteen hour exposure of medfly resulted in the same effect as observed for 7 h. As the sticking of the flies to the ground prevented the unambiguous association of death with the H_2_O_2_ treatment, the experimental setup for further experiments was changed to mosquito cages lined with paper on the floor (Supplementary Figure 2E). *D. melanogaster, Sp. pandurus* and *T. castaneum* were still unaffected by the extended treatments.

### Lethal Exposure Times for Mosquitoes, Medfly, and *Drosophila* at 400 ppm H_2_O_2_

**FIGURE 3:**
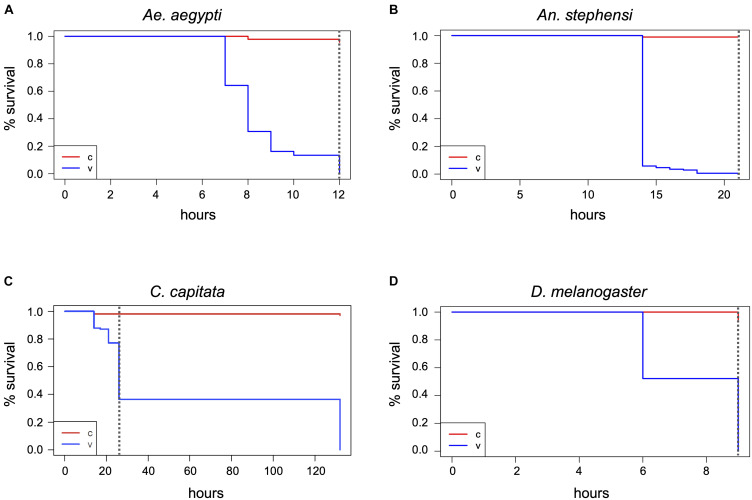
Determination of the maximum lethal exposure time for mosquitoes, medfly, and *D. melanogaster*. *Ae. aegypti* **(A)**, *An. stephensi* **(B)**, *C. capitata* **(C)**, and *D. melanogaster* in the absence of food **(D)** were continuously exposed to 400 ppm hydrogen peroxide and viability assessed at regular intervals until the last individual was dead. The Kaplan-Meier curves show the probability of survival per time interval for the treatment (v) and the control (c). The dashed vertical line in the graphs indicates the end of the H_2_O_2_ exposure. Survival numbers from all biological replicates per species were combined for the analysis. Data shown are based on three biological replicates for the mosquitoes (v and c) and the medfly control, two for the *Drosophila* and medfly treatment, and four for the *Drosophila* control.

Based on the above results, the lethal H_2_O_2_ treatment time necessary to kill all *Aedes* mosquitoes (LT_100_) is between 7 and 14 h, whereas the LT_100_ for *Anopheles* mosquitoes is between 14 h and 21 h. To determine the exact LT_100_ for both species, one cage (65–90 individuals) and three vials per species (25–40 individuals each) were exposed to 400 ppm H_2_O_2_ and checked hourly. The LT_100_ for *Ae. aegypti*, both in cages and vials, was between 10 and 12 h (Figure 3A and Supplementary Table 1). The LT_100_ for *An. stephensi* in the better aerated cage was 15 h, whereas the last insects died in the vials between 16 and 21 h (Figure 3B and Supplementary Table 1). For both species, the number of deaths during the treatment was significantly different from the controls (*p* < 2^–16^; Chi-square test, based on combined survival numbers of vials and cage).

Using the adjusted cage setup, fresh cohorts of medflies were exposed to 400 ppm H_2_O_2_ for 26 h. After 14 h, several individuals were supine, others were crawling, but none were able to fly, and 17 of 140 flies were dead (Figure 3C and Supplementary Table 1). After 26 h, 55–60% of the flies had died, compared to none of the control flies (*p* < 2.2^–16^). The survivors of the 26-h treatment were affected, some showing minimal movement, others crawling but unable to fly and often falling into a supine position. All flies were dead 4.5 days after the end of the treatment despite supply of food and water, while in the controls only three flies died during the whole period (Chi-square test, *p* < 2^–16^). The treated flies showed evidence of H_2_O_2_ damage, such as a frosted carapace and dulled eyes (not shown). The medfly LT_100_ could not be determined as the airlock was not available for longer treatments.

As it could not be excluded that the fly food in the *Drosophila* rearing containers (Supplementary Figure 2F) changed the effect of the H_2_O_2_ on the flies, exposure was repeated with two fresh cohorts of flies in empty vials covered with mesh (Supplementary Figure 2A). In the absence of food all flies were dead within 9 h (Figure 3D and Supplementary Table 1). None of the 96 control flies outside the airlock (C1–C3, Supplementary Table 1) died during this time. To exclude death in the treatment cohorts due to the overall more stressful environmental conditions within the airlock (temperature, humidity, and lack of food), additional control incubations were performed in the laboratory under conditions closer to the H_2_O_2_ treatment [27°C, 70% RH (C6, C7), and 28°C, 37–41% RH (C4, C5), all without food] with similarly aged flies. Here, only 5% were dead after 9 h (Supplementary Table 1), showing that the 100% mortality of flies exposed to 400 ppm H_2_O_2_ for 9 h was statistically significant (Chi-square test, *p* < 2^–16^) and related to the fumigant rather than the elevated temperature or RH.

### Milkweed Bugs and Flour Beetles Show Moderate or No Effects of Extended H_2_O_2_ Exposures

**FIGURE 4:**
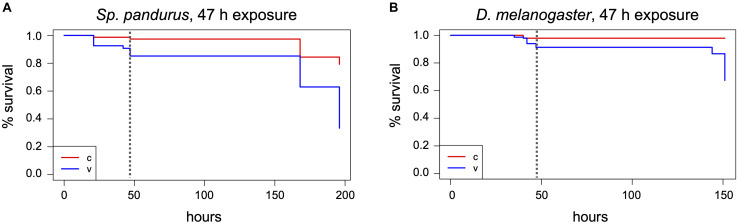
Effects of long-term H_2_O_2_ exposure on the milkweed bug and *Drosophila*. *Sp. pandurus* nymphs and adults **(A)**, and *D. melanogaster* adults **(B)** were exposed to 400 ppm H_2_O_2_ for up to 47 h and death assessed at intervals. The Kaplan-Meier curves show the combined probability of survival per time interval for the treatment and post-treatment monitoring time, as described in Figure 2. The dashed vertical lines indicate the end of H_2_O_2_ exposure. Data shown here is based on two biological replicates for *Sp. pandurus* and five biol. replicates for *D. melanogaster*; v, treatment; c, control.

*Spilostethus pandurus* and *T. castaneum* containers were subjected to an extended 400 ppm H_2_O_2_ treatment for a total of 47 h. In the *Sp. pandurus* containers, two small larvae had died after 21 h exposure, both showing a pink to light red color that is apparent immediately after ecdysis, when the chitin shell has not yet hardened and darkened. One larger larva had died during ecdysis while still attached to the old chitin shell. All other individuals showed no visible effects. At the termination of the exposure eight of the original 54 *Sp. pandurus* larvae were dead (compared to two of 77 control specimens, *p* = 0.01275, Chi-square test), but the survivors appeared less lively than the control insects. This indicated physical but non-lethal damage induced by the treatment. Further monitoring for 7 days confirmed this observation, resulting 36 deaths in the treatment groups, compared to 16 of the 77 control insects (*p* = 2^–7^, Figure 4A and Supplementary Table 1).

Remarkably, not a single beetle was dead in the *Tribolium* vials (experimental or control) after exposure to 400 ppm H_2_O_2_ for 47 h and 7 days post-treatment. All individuals were lively and highly viable, showing not the slightest visible effect of the treatment (Supplementary Table 1).

### H_2_O_2_ Absorption by *Drosophila* Fly Food Neutralizes the Effect on Adult Flies but Kills Embryos Deposited in the Food

During the H_2_O_2_ exposures in the presence of fly food, it was observed that adult *D. melanogaster* flies remained mostly on the food surface and the filter paper placed into the food. To test if the moisture in the food and filter paper may have protected the flies by absorbing the reactive molecules, H_2_O_2_ indicator strips were placed in *Drosophila* food vials without insects at different positions relative to the food surface. Empty vials with the indicator strip at the bottom served as controls (Supplementary Figure 3A). These test vials were exposed to 400 ppm H_2_O_2_ for 1 h followed by 1 h aeration. During the exposure it was observed that the indicator dye changed its color faster in the empty vials (Supplementary Figures 3B,C), displaying yellow color at the end of the treatment. The degree of color change in the vials with food depended on the position of the indicator spot relative to the food surface (Supplementary Figures 3C–E). Indicator spots positioned partially into a crack in the food changed color only from dark to light purple, while spots placed directly above the food surface changed towards orange (Supplementary Figures 3D,E). This indicates that the fly food indeed neutralized the effect of the reactive H_2_O_2_ molecules and thus protected the flies. Correspondingly, even extended exposures of 40 h (V4 and V5) or 47 h (V1-V3) in the presence of fly food resulted only in low death rates of 5–10% (compared to 3% in the controls; *p* = 2^–8^; Chi-square test, combined numbers of vials 1–5; Figure 4B and Supplementary Table 1).

H_2_O_2_ is a weak acid (p*K*_a_ = 11.75), a powerful oxidizing agent, and can form free hydroxide radicals. If H_2_O_2_ reactive molecules are absorbed by the food, it might affect the viability of the *D. melanogaster* eggs laid into the food. To verify this, flies were removed from the treated and control vials 5 days after the last H_2_O_2_ treatment (10 days after the initial transfer of adults to the vials), and larval development was assessed. Normal larval feeding activity was observed in the control vials, but no feeding traces were visible in any exposed vials. A few early instar larvae were visible on the food surface in V7 (high ppm treatment), but they all appeared dead. The food color was lighter in the exposed vials, suggesting H_2_O_2_ bleaching. Thirteen days after the last H_2_O_2_ treatment, 1–4 freshly emerged adults and many pupae were counted in the control vials. The food in V7 showed very few and faint traces of larval feeding activity, but no larvae were found. Four weeks after the last H_2_O_2_ treatment, a single pupa was observed in V7, but still no traces of larvae or pupae in any of the other treated vials. These results indicated that the H_2_O_2_ reactive molecules indeed killed most embryos embedded in the food or harmed their larval development.

### Increased H_2_O_2_ Concentration Kills Mosquitoes Quickly

**FIGURE 5:**
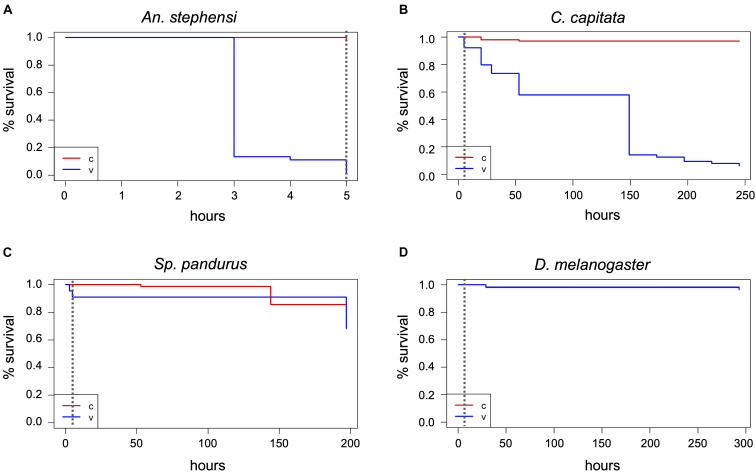
Lethality induced by short-term exposure to 1000 ppm H_2_O_2_. Insects were continuously exposed to 1000 ppm hydrogen peroxide for up to 5 h and death assessed in intervals. Survivors were monitored for another 5–8 days after treatment end and death assessed in intervals. Data analysis shown here was performed as described in Figure 2 and is based on one large experimental cohort for *An. stephensi* **(A)**, *C. capitata* **(B)**, and *Sp. pandurus* **(C)**, and two biological replicates for *D. melanogaster* **(D)**. The dashed vertical lines indicate the end of the H_2_O_2_ exposure; v, treatment; c, control.

Given that several species appeared unaffected or only mildly affected by prolonged exposure to 400 ppm H_2_O_2_, the effect of a short-term (5 h) exposure to 1000 ppm on all test species was investigated. Again, the mosquitoes were most sensitive to the treatment. All the *Ae. aegypti* specimens were killed and bleached within 3 h, the *An. stephensi* after 5 h, with 90% dead after 3 h (Figure 5A and Supplementary Table 1). In contrast, only ∼10% of the *C. capitata* specimens were dead after 5 h, although the survivors showed the same strongly impaired fitness as observed for the extended 400 ppm exposure. Six days post-treatment, only nine of the original 64 flies remained alive, significantly less than in the controls (*p* < 2^–16^) (Figure 5B). As in the 400 ppm treatment, two *Sp. pandurus* larvae died during or after ecdysis when the chitin carapace is softer and contains more moisture, probably enhancing sensitivity to chemical burns. Compared to the long-term treatment, the short exposure to 1000 ppm H_2_O_2_ did not affect the bugs’ fitness, as the death of treated individuals was not significantly different from the controls in the post-treatment monitoring period (Figure 5C; *p* = 0.3). The high ppm exposure of *Drosophila* was again conducted in standard *Drosophila* rearing vials, including fly food. As in the previous experiments in the presence of food, no fly died during the exposure to 1000 ppm H_2_O_2_ (Figure 5D). Similarly, all *T. castaneum* specimen survived without any trace of damage (Supplementary Table 1).

### H_2_O_2_ Reduces the Viability of *Aedes* Eggs in a Dose-Dependent and Time-Sensitive Manner

Matured *Ae. aegypti* eggs show strong desiccation resistance and can survive immersion in 30% sodium hypochlorite (NaOCl) for at least 30 min (Rezende et al., 2008). This is attributed to a combination of melanization and the formation of a thin but sturdy serosal cuticle underneath the chorion between 11 and 13 h after egg laying (Rezende et al., 2008; Farnesi et al., 2017). To investigate the sensitivity of mature *Ae. aegypti* eggs to gaseous H_2_O_2_, we exposed eggs to 400 ppm H_2_O_2_ for 7, 14, and 21 h, and to 1000 ppm H_2_O_2_ for 5 h.

**FIGURE 6:**
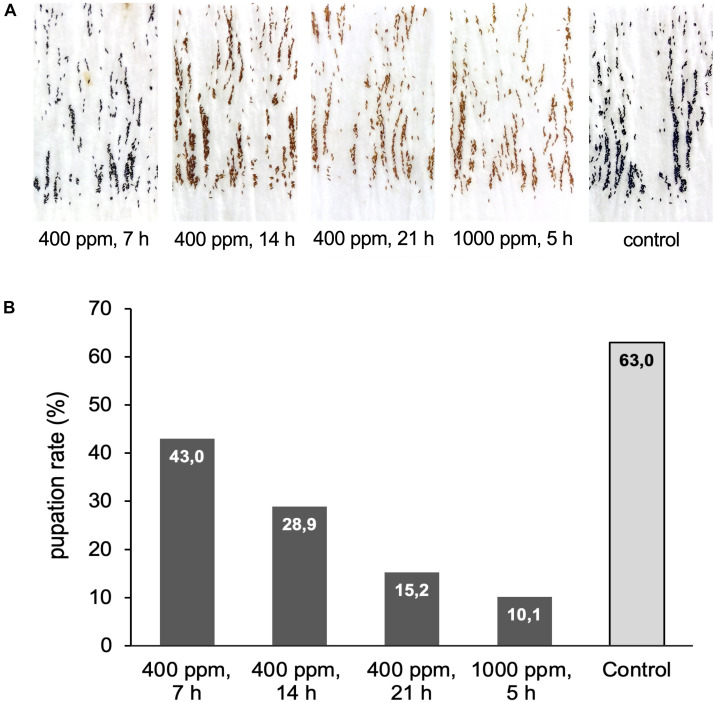
Exposure of *Ae. aegypti* eggs to hydrogen peroxide. **(A)** Pictures of *Ae. aegypti* eggs displaying different degrees of bleaching after exposure to 400 ppm H_2_O_2_ for 7, 14, or 21 h, and to 1000 ppm for 5 h. The control batch was kept next to the airlock for the duration of the exposure. **(B)** Eggs from **(A)** were submerged in water to assess the influence of the H_2_O_2_ treatment on egg viability. Shown is the pupation rate (number pupae/number eggs*100) for the different treatments. Data are based on one large experimental cohort of more than 1000 eggs for each treatment.

The 7 h exposure at 400 ppm left no visible effects on the eggs compared to the untreated controls. After 14 h the eggs showed slight bleaching that became more severe after 21 h and at the high ppm exposure (Figure 6A). The hatching of all egg samples was rapid and spontaneous, but the larvae in the 1000 ppm treatment group showed a high mortality rate during the first 5–24 h after hatching, with more than 200 of the 1800 larvae dead after 24 h. A few dead larvae (∼30) were also observed in the 21 h 400 ppm H_2_O_2_ treatment group, but the effects at this dose were less severe compared to 1000 ppm. No early larval mortality was seen for the shorter exposure times or the untreated control. Larval development in the different treatment groups was delayed in accordance with the larval mortality data. In the control egg batch and the eggs exposed to 400 ppm H_2_O_2_ for 7 and 14 h, the larvae reached stage L3 4 days after hatching. In the 21 h exposure, the larvae had reached stage L2 by the same time, and in the cohort exposed to 1000 ppm H_2_O_2_ larvae were between L1 and L2 stage. The pupation rate (compared to the number of eggs) decreased almost linearly with increasing H_2_O_2_ exposure time and concentration, from a maximum of 63% in the control group to a minimum of 10% in the cohort exposed to 1000 ppm H_2_O_2_ (Figure 6B).

### Exposure at 50°C Is an Effective Method for the Fast Inactivation of Insects

As an alternative to H_2_O_2_ exposure, the possibility of emergency inactivation of insects by heat was investigated. It would technically be possible to heat a medium-sized room of 15–20 square meters, such as a BSL-3 insectary, to a maximum of 60°C. Heat sensitivity of insects representing different orders (*D. melanogaster*, *C. capitata*, *Ae. aegypti*, and *T. castaneum*) was tested by incubating insects individually at temperatures between 25°C (control) and 50°C for up to 72 h. Preliminary experiments showed that the insects sometimes were knocked down at the end of the heat treatment but recovered within several hours. Therefore, for each exposure temperature and duration, a new experimental cohort (20 individuals) was used, and all individuals were regularly checked for recovery or death for 24 h post-treatment. Incubations at each temperature and time interval were repeated at least twice. For *Aedes* only females were investigated due to their higher resilience to general stress factors like temperature or lack of water as observed in daily rearing routines.

**FIGURE 7:**
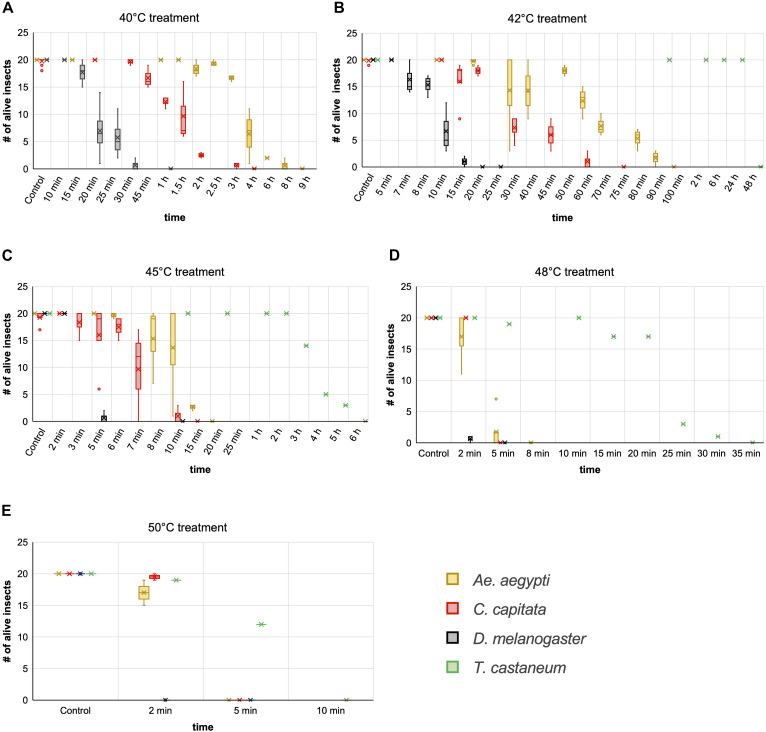
Comparison of insect heat tolerance at five different temperatures. Cohorts of 20 insects were exposed to 40°C **(A)**, 42°C **(B)**, 45°C **(C)**, 48°C **(D)**, and 50°C **(E)** for increasing time periods. For every combination of temperature and time a fresh cohort was used, and death was monitored for 24 h post exposure. Box and whisker charts are showing the inclusive median of survival numbers of the insects depending on the treatment temperature and exposure time. The means are indicated by an x, outliers are displayed as dots. Except for T. castaneum, the survival numbers shown here for every combination of temperature and treatment time are based on two to five repetitions with 20 individuals each. The survival numbers displayed for the controls are based on three to 12 repetitions with 20 individuals each.

Of the three dipterans, *Ae. aegypti* showed the strongest heat tolerance. Individual females survived exposure to 40°C for up to 8 h. The LT_100_ at 40°C was determined to be 9 h. In comparison, lethal incubation times for *C. capitata* and *D. melanogaster* at 40°C were 4 and 1 h, respectively (Figure 7A). While *Ae. aegypti* survival times strongly decreased at higher temperatures, to a maximum of 90 min at 42°C, 15 min at 45°C, and 5 min at 48°C, they were in all cases markedly higher than for medfly and *Drosophila* (Figures 7B–D). At the highest tested temperature, 50°C, *Aedes* and medfly could withstand a 2-min incubation without problems but were dead after 5 min. All *Drosophila* specimen died during the 2-min exposure. As observed for the H_2_O_2_ treatments, *T. castaneum* was again the most resilient species, with more than half of the cohort withstanding 50°C for 5 min and displaying longer survival times at all other temperatures (Figures 7B–E). According to these results, 50°C was defined as lethal temperature for all tested species that kills within a few minutes. The exact survival numbers for all species are provided in Supplementary Table 3.

**FIGURE 8:**
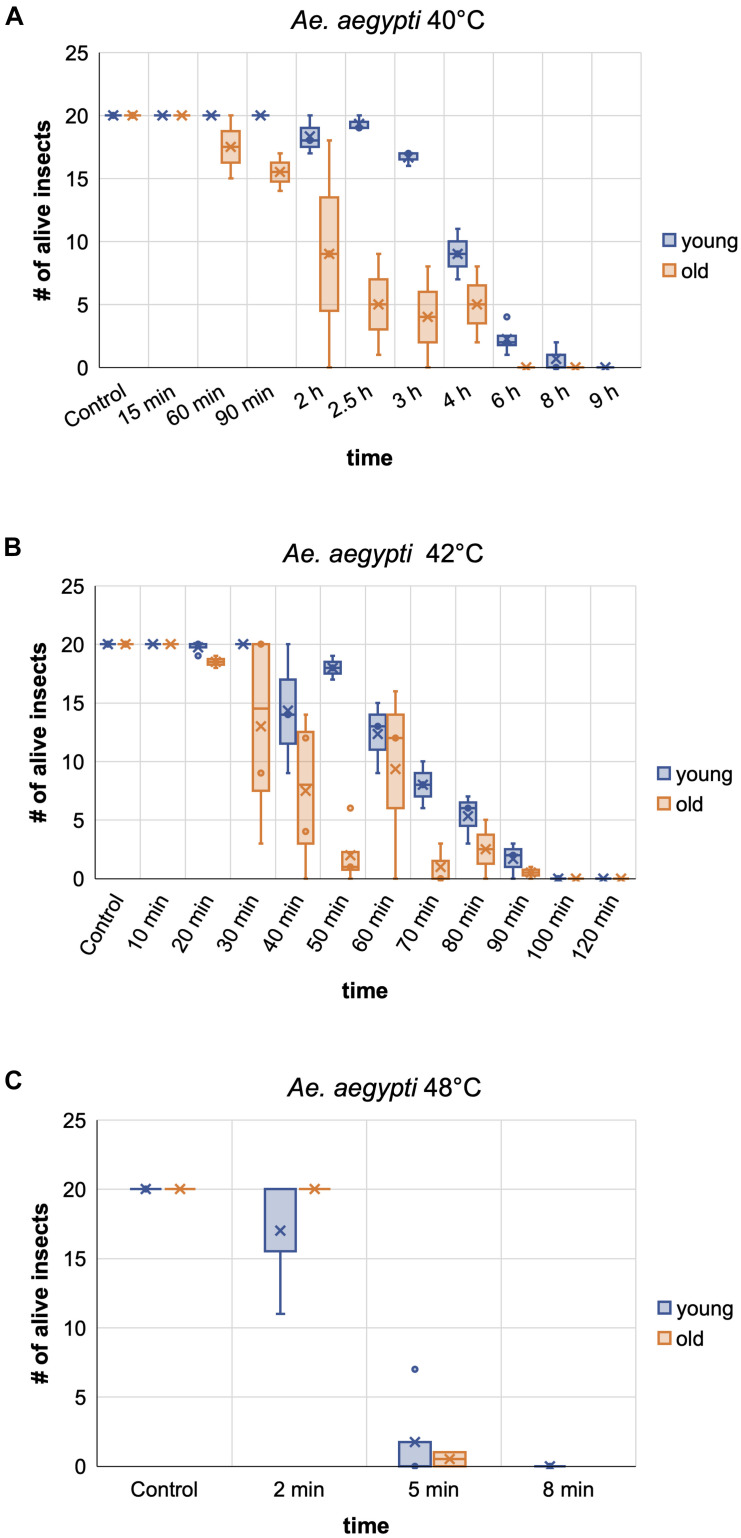
Comparison of the heat tolerance of *Ae. aegypti* female mosquitoes at different age. Influence of age on heat sensitivity was investigated at two moderately increased temperatures, 40 and 42°C **(A,B)**, and at a near-lethal temperature, 48°C **(C)**. “Young” females were between 1 and 10 days old, “old” females between 10 and 29 days. Lethality for each exposure temperature and time was assessed in two to four biological replicates (i.e., each replicate was performed with an experimental cohort from a different cage). Each cohort consisted of 20 individuals. The box and whisker charts display the inclusive median of the replicate values for each exposure time. The means are indicated by an x, outliers are shown as dots. The survival numbers displayed for the controls are based on two to eight repetitions with 20 individuals each.

We moreover selected *Ae. aegypti* to test the influence of the insects’ age on temperature resilience, using mainly five different age groups of females; 1–7, 5–10, 10–14, 15–17, and 18–29 days post emerging. Based on the survival data from the above experiments, 40 and 42°C were selected as less stringent temperatures for the assay and 48°C as near-lethal temperature. The results obtained from the different age groups indicate that around day ten post emerging is a critical age for the heat tolerance of *Ae. aegypti* females at moderately elevated temperatures (40 and 42°C). At short to intermediate incubation times, females younger than 10 days showed markedly higher heat resilience than females older than 10 days (Figures 8A,B). When approaching the near-lethal incubation time for both temperatures, the survival numbers of “young” and “old” females converged, resulting in similar LT_100_ timepoints. Interestingly, the survival data recorded for the “young” age groups (1–7 and 5–10) at 40 and 42°C displayed minimal variance between the biological replicates, while the survival times of the older age groups (10–14, 15–17, and 18–29 days) varied strongly between replicates (Figures 8A,B). This variation, however, could not unambiguously be linked to the age difference between the “old” age groups, as in several replicates the oldest females were more resilient than younger ones (Supplementary Table 2). No age-related difference in heat sensitivity could be observed at 48°C (Figure 8C).

## Discussion

We tested if either H_2_O_2_ fumigation or heat treatment has the potential to be developed into a standard procedure for the safe and ethical room-wide decontamination of BSL-3 insectaries in emergency situations, such as mass escapes of pathogen-infected insects. To account for the diversity of vector insects potentially held in BSL-3 insectaries, the effectiveness of both methods was tested against insect species representing different orders.

This study shows that heat is a very effective way to kill a broad range of species, with a temperature of 50°C sufficient to kill all specimens within 10 min under the chosen experimental conditions. Also, 48°C effectively killed in less than 1 h. These values are in accordance with studies in the early 1900s, showing that 48–50°C are effective to kill different stored product insect pests within a few minutes (Dean, 1913). Translating these results to a room-wide procedure will require adaptation of treatment times, as the walls, furnishing, and equipment will delay heating to the target temperature. Required time and energy can be estimated using the specific heat capacity and approximate volume of all the materials (assuming perfect room insulation without heat loss to the surrounding areas). Like for room decontaminations by H_2_O_2_ fumigation, however, safe treatment times will have to be validated for each specific facility and species. Temperatures lower than 48°C are not recommended, as even under laboratory conditions, extended treatment times of several hours were required to reach LT_100_. Moreover, treatments at sub-lethal temperatures can increase the risk of heat acclimation, which could decrease treatment efficacy. Such acclimation has been shown for *T. castaneum* already after 5 h at 42°C (Lu and Liu, 2017). Given a slow overall room heating rate and material-dependent uneven heat-distribution, such acclimation conditions could be created during the heating of a BSL-3 insectary, if for technical or financial reasons the planners would favor long incubation times at lower temperatures over short treatments at high temperatures, and if no fans are used to achieve uniform heating rates across the room.

The pronounced heat tolerance observed for *T. castaneum* in this study has been known for years and was linked to adaptation mechanisms, including the upregulation of heat-shock genes like *hsp70*, which is strongly upregulated during heat acclimation at 42°C (Lu and Liu, 2017). Lethal incubation times at 50°C observed in this study are similar to published values (Fields, 1992; Mahroof et al., 2003; Yu et al., 2017). Variations between the different studies might be due to the experimental setup [size and material of the container and the presence of other heat-absorbing materials delaying the heating to the target temperature (e.g., flour)], and the age of the adult beetles.

Our study also revealed a strong difference in tolerance to moderately elevated temperatures (40–42°C) between the three dipterans, which could be related to the species’ adaptation to their natural habitats in tropical and subtropical areas (*Aedes* and medfly), or in temperate climates (*D. melanogaster*). In addition, tolerance against dehydration might play a role, which in part is influenced by the insects’ body size, making the small *Drosophila* the most susceptible species. While we did not control for humidity in our experimental setup, studies with different insects suggest that controlling for low humidity during the room-wide heat treatment can improve the efficacy (Mellanby, 1932; Denlinger and Yocum, 1999; Ginsberg et al., 2017). The reasons for the strong variation in temperature tolerance of *Aedes* females older than 10 days are unclear. It has been shown, however, that nutrient accumulation, density, and temperature during larval development can influence adult quality and life history traits (Couret et al., 2014; Hapairai et al., 2014; Yahouedo et al., 2014; Oliver and Brooke, 2017; Puggioli et al., 2017), and the tolerance of male *Ae. aegypti* to heat stress (Sasmita et al., 2019). Similar to *T. castaneum*, it was moreover observed for *Aedes* that pre-exposure of larvae to sub-lethal temperatures can confer adaptive thermotolerance (Patil et al., 1996). *Aedes* mosquitoes used in this study were grown at a constant temperature of 27 ± 0.3°C, and larvae and adults were fed with standardized food, excluding an effect of these factors on variations in temperature tolerance. However, our standard rearing protocols do not control for exact larval densities by counting, but instead, make a visual assessment of the density in the rearing trays and feed the larvae *ad libidum*. Therefore, the amount of food per larva can vary between larval rearing cohorts, which could lead to variations in nutrient accumulation during larval development. This might have influenced the heat tolerance of the “old” females in replicates performed with different rearing cohorts.

In contrast to the heat treatments, it was impossible to determine suitable H_2_O_2_ fumigation conditions applicable to all tested species in a room-wide scenario. Our results suggest that the thickness of the chitinous exoskeleton might play a substantial role in the effects of H_2_O_2_ fumigation, as species with a thinner exoskeleton (particularly *Drosophila*, *Aedes*, and *Anopheles)* were much more sensitive than species with a thick exoskeleton like the milkweed bug and flour beetle, with the latter appearing to be insensitive to the applied H_2_O_2_ concentrations and treatment times. Higher H_2_O_2_ doses are not applicable for several reasons: first because high concentrations (≥1000 ppm) would lead to condensation of the gas on surfaces, which could damage furnishing and equipment, especially metals could be oxidized, and second, because large quantities of H_2_O_2_ would be required for a room-wide application over many hours. The H_2_O_2_ exposure results obtained for the small dipterans, predominantly the mosquitoes, can probably be transferred to other vector insects of similar physical constitution like phlebotomine sandflies. Similarly, the results obtained for the bugs and beetles in our study might be extendable to ticks, which also have a thick exoskeleton and can therefore be expected to be essentially insensitive to H_2_O_2_ fumigation.

In summary, this study shows that H_2_O_2_ fumigation would only be applicable to a subset of the vector insects typically held in BSL-2 or BSL-3 insectaries. Its applicability as room-wide emergency decontamination would have to be evaluated for each new insect species added to the laboratory, which, in our opinion, prevents H_2_O_2_ fumigation from being established as a standard procedure for insect inactivation in BSL-3 insectaries. In contrast, the combined results of the heat treatments suggest 50°C to be a reliable and universally applicable temperature to quickly and irreversibly inactivate insects of all tested species. The results are probably also applicable to related species and those with similar physical properties, unless they have a specific heat tolerance due to adaptation to high temperature conditions in their natural habitats. Therefore, heat treatment has the potential to be developed as a standard and commonly recognized room-wide insect emergency inactivation method.

## Data Availability Statement

The original contributions presented in the study are included in the article/Supplementary Materials, further inquiries can be directed to the corresponding author/s.

## Author Contributions

IH designed the experiments. IH, RK, JM, EL, JR, and TR performed the experiments. IH and GE analyzed the data. IH, GE, and MS wrote the manuscript. All authors contributed to the article and approved the submitted version.

## Conflict of Interest

RK was employed by the company Ortner Reinraumtechnik GmbH. The remaining authors declare that the research was conducted in the absence of any commercial or financial relationships that could be construed as a potential conflict of interest.
