# Supplementary material for: Evaluation of Hydrogen Peroxide Fumigation and Heat Treatment for Standard Emergency Arthropod Inactivation in BSL-3 Insectaries
Source: Front Bioeng Biotechnol. 2020 Nov 16;8:602937. doi: 10.3389/fbioe.2020.602937 (PMC7701145; doi:10.3389/fbioe.2020.602937)
Supplement: Supplementary file 1 [file Data_Sheet_1.pdf]

Supplementary material

Contents:

Suppl. Figure 1 - Hydrogen peroxide concentration curves and experimental cohorts per run

Suppl. Figure 2 - Experimental cages used for the hydrogen peroxide treatments

Suppl. Figure 3 - *Drosophila* food reduces the hydrogen peroxide effects

Suppl. Table 1 - Hydrogen peroxide treatment conditions and insect survival numbers

Suppl. Table 2 - Survival numbers of *Ae. aegypti* females upon heat exposure

Suppl. Table 3 - Survival numbers of heat treatments.

A

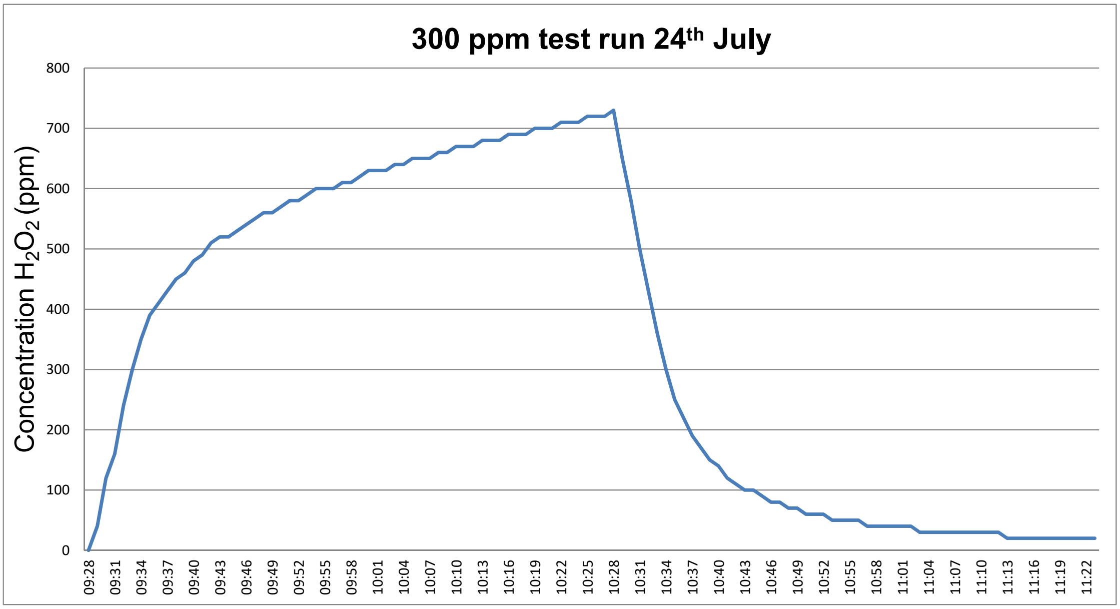

Run 1: First test run with *Ae. aegypti* and *T. castaneum* for one hour, performed on July 24th. Target H_2O_2 concentration: 300 ppm; the run was stopped after 1 h by aeration of the airlock. y-axis displays the H_2O_2 concentration measured by the control unit of the airlock, x-axis the time of the day.

B

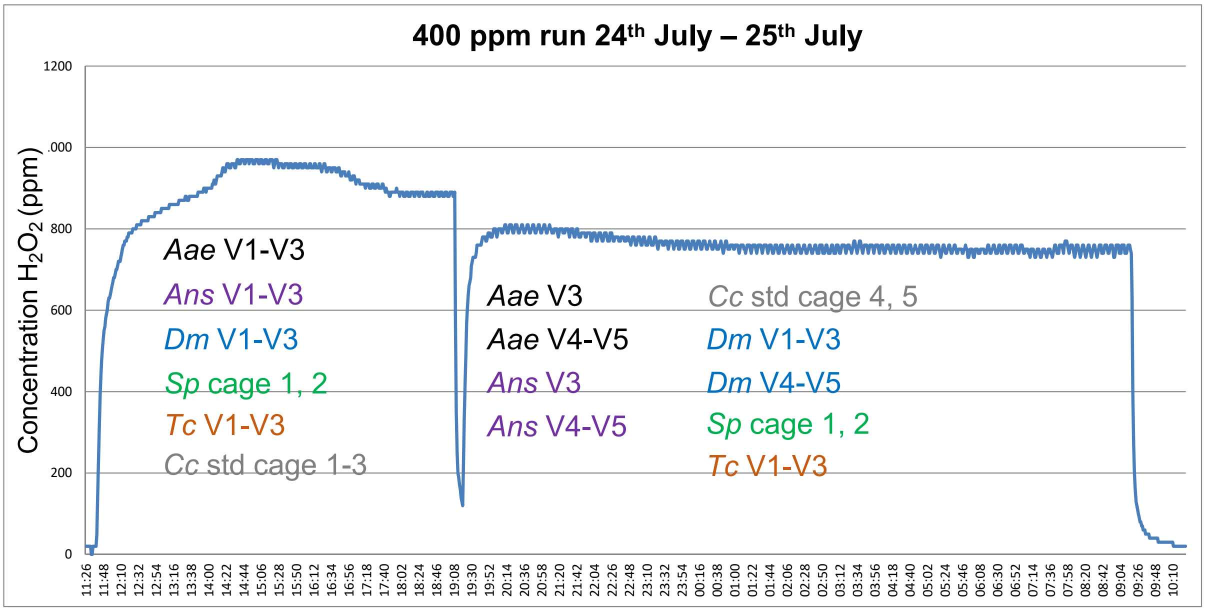

Run 2: Subsequent run with all test species from July 24th at 12:00 (noon) until July 25th, 9:30 (am). Target H_2O_2 concentration: 400 ppm (ramp time to reach target concentration = 30 min); the airlock was opened between 19:00 and 19:30 on July 24th to assess the survival status of all test and control individuals and for the removal of Aae and Ans V1 and V2, and Cc cages 1-3. All other cages were returned to the airlock for continuation of the treatment overnight (21 h treatment). Moreover, new containers were added at this timepoint (14 h treatment); y- and x- axis as in A)

Supplementary Figure 1. Hydrogen peroxide concentration curves and experimental cohorts per run. Shown are the H_2O_2 concentration curves recorded by the airlock during the different runs and the respective experimental cages exposed to H_2O_2 during each run. Due to constraints in the availability of the airlock and the extended duration of the experiments, some of the experiments had to be performed in parallel. All details of the experiments are given in the run descriptions. *Aae, Ae. aegypti; Ans, An. stephensi; Cc, C. capitata; Dm, D. melanogaster; Sp, Sp. pandurus; Tc, T. castaneum.*

C

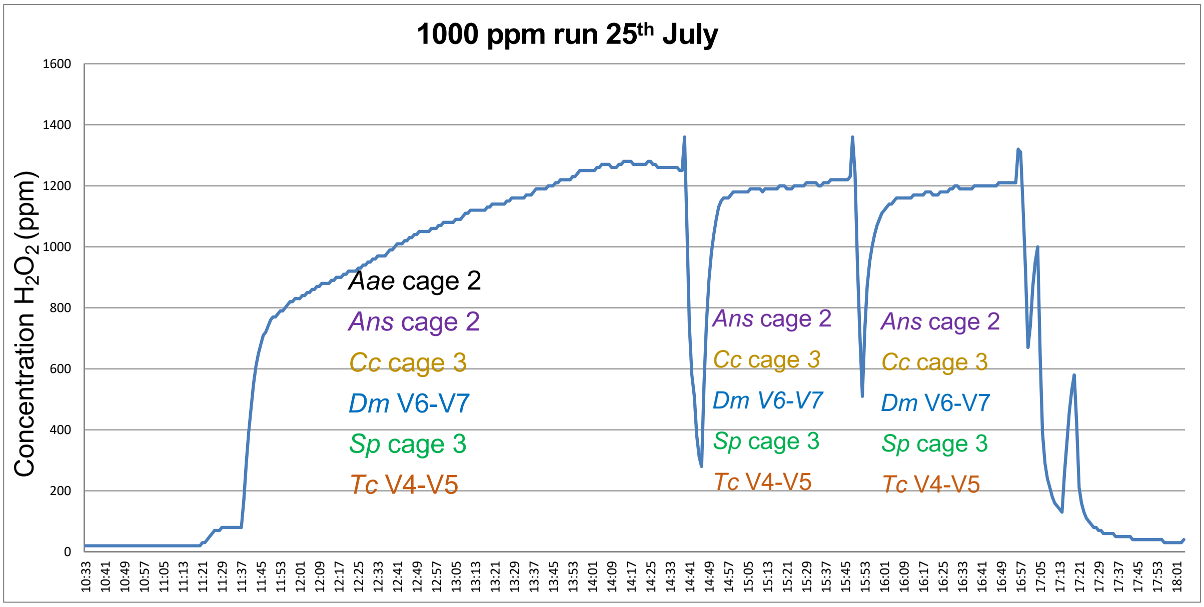

Run 3: After assessment of the 14 and 21 h overnight treatments from run 2, the long-term 400 ppm treatment cohorts were stored at ambient conditions, and a high ppm run with fresh individuals of all test species was performed on July 25th. Target H_2O_2 concentration: 1000 ppm (ramp time to reach target concentration = 30 min); the airlock was opened after 3 h, 4 h and 5 h to assess the survival status of all test individuals. The run was terminated after 5 h of treatment. y- and x- axis as in A)

D

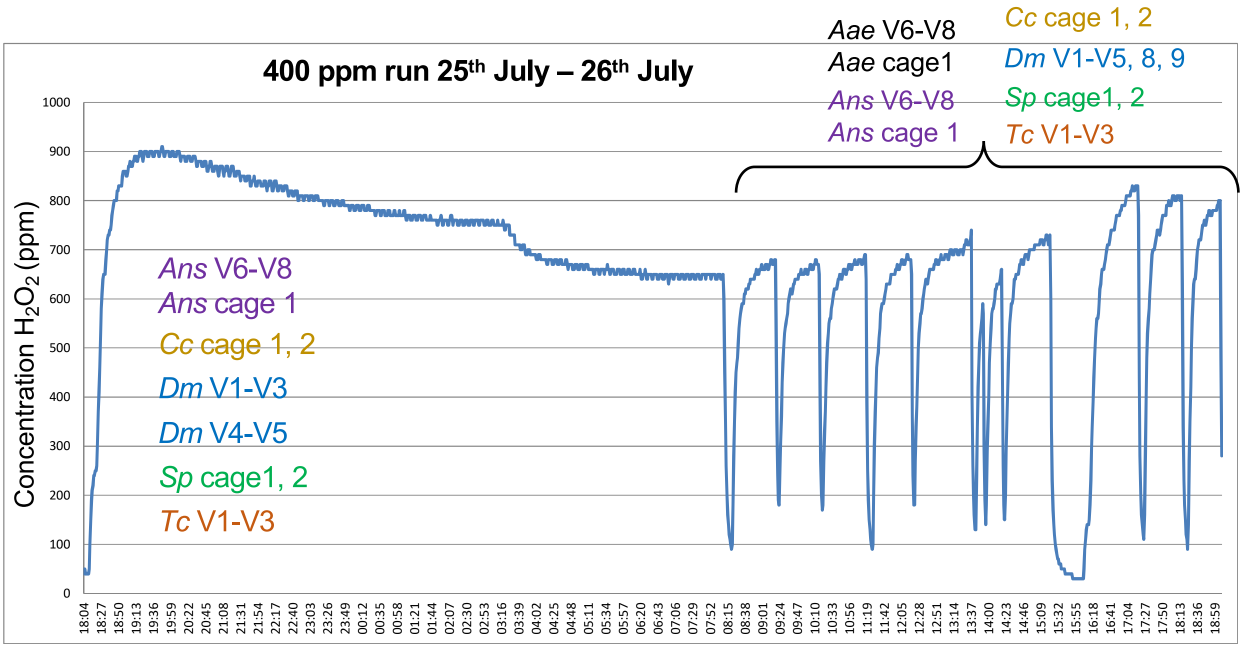

Run 4: After termination of the high ppm run (run 3), the long-term 400 ppm treatment was continued with the individuals from run 2. Insects had been stored at ambient conditions in the meantime. Target H_2O_2 concentration: 400 ppm (ramp time to reach target concentration = 30 min); Moreover, the critical exposure time experiments for *Aedes, Anopheles,* and *Drosophila* w/o food were performed during this run. For this purpose, the airlock was opened every 60 min on July 26th to assess mosquito and *Drosophila* death. y- and x- axis as in A)

Supplementary Figure 1 (continued). Hydrogen peroxide concentration curves and experimental cohorts per run.

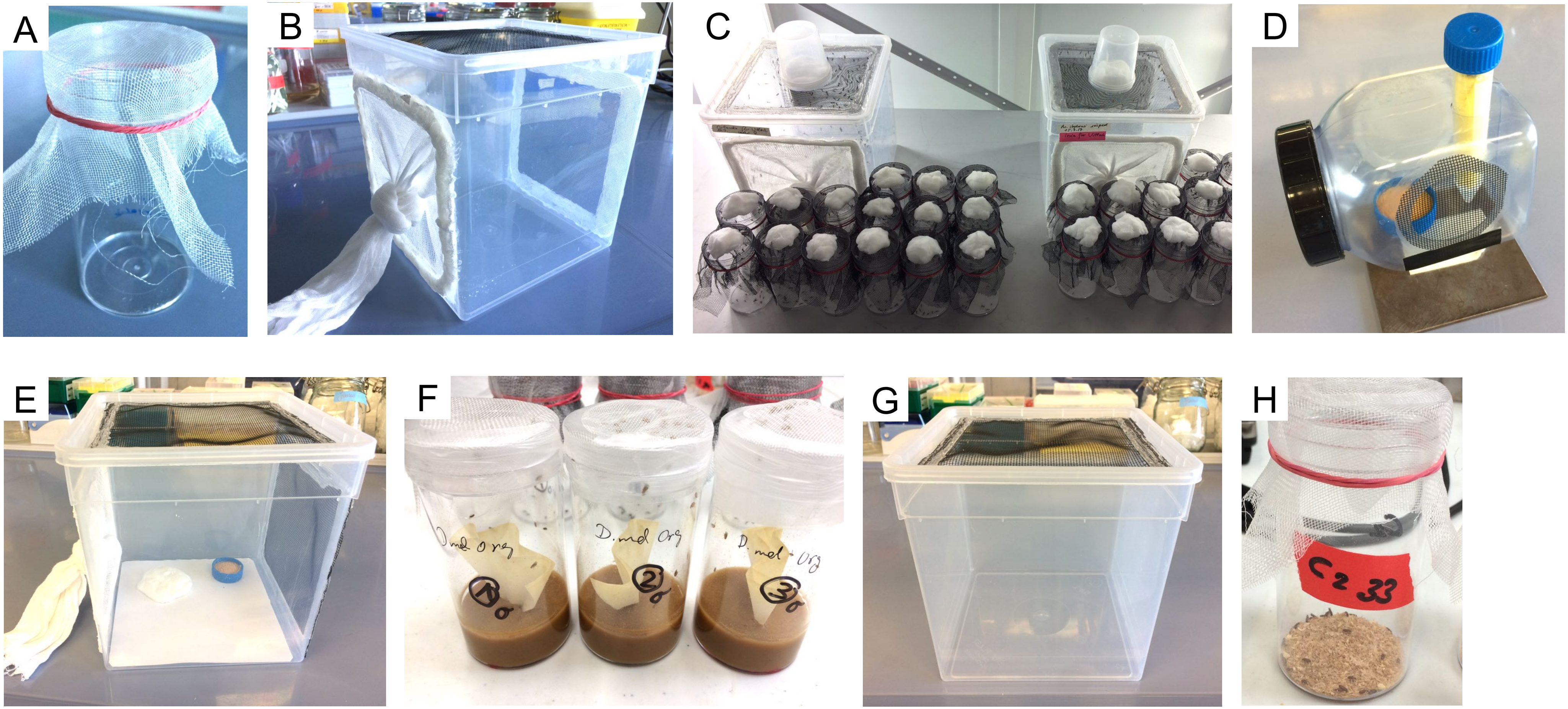

Supplementary Figure 2. Experimental cages used for the hydrogen peroxide treatments. (A) empty *Drosophila* vial covered with mesh used for mosquitoes, *T. castaneum*, and the *D. melanogaster* w/o food experiments; **(B)** empty cage used for mosquitoes; **(C)** mosquito experimental containers supplied with sugar-soaked cotton; **(D)** Medfly rearing cage used for the initial experiments (7 and 14 h at 400 ppm); **(E)** Medfly experimental cage with food and water supply, lined with paper on the bottom to prevent sticking (used for 5h at 1000 ppm and 26 h at 400 ppm); **(F)** *D. melanogaster* rearing vials with food, used for all experiments except the lethal exposure determination experiment (Figure 3D); **(G)** Cage used for *Sp. pandurus*; **H)** experimental vial with *T. castaneum*; the bottom is covered with a thin layer of sawdust and traces of wheat flower

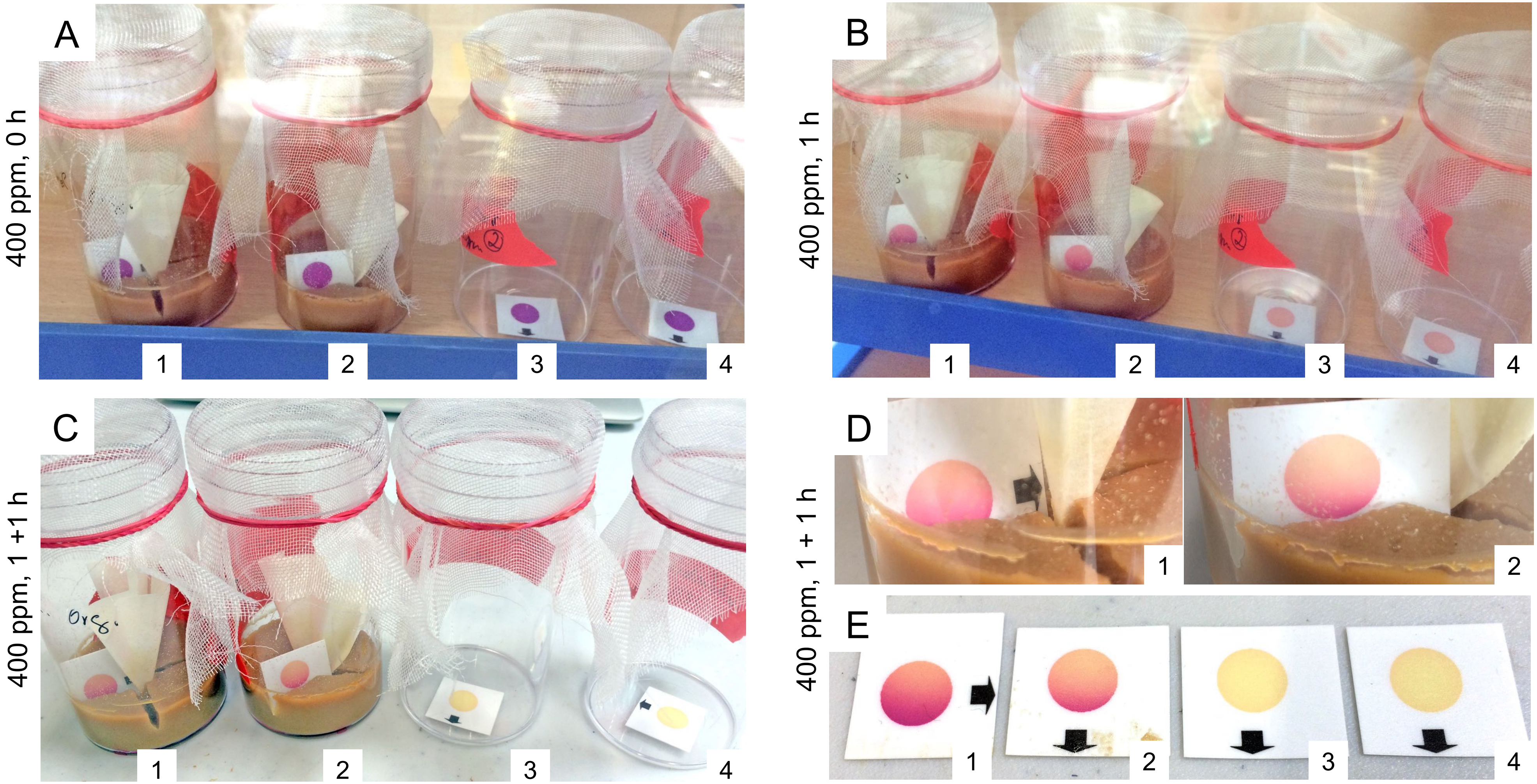

Supplementary Figure 3. *Drosophila* food reduces the hydrogen peroxide effects. Steraffirm VH2O2 indicator strips were placed into *Drosophila* food vials without flies (1, 2), and into two empty vials as a control (3, 4). Vials were exposed to 400 ppm for 1 h, then the airlock was aerated for 1 h. **(A)** indicator strips at the start of the experiment. **(B)** indicator strips after 1 h exposure, before start of the aeration. The strips in the control vials (3, 4) already show slightly lighter coloring than the ones in the food vials. **(C)** Strips after conclusion of the experiment (2 h total). The strips in the control vials have turned yellow. **(D)** close-up view of the two indicator strips placed into the drosophila food. The strips were placed into cracks in the food at different depths, such that the lower edge of indicator spot was either below the surface level of the food (1), or just at the same level as the food surface (2). **(E)** Indicator strips taken out of the *Drosophila* vials after the treatment. The strips in the control vials turned to even yellow color (3, 4), whereas the indicators in the food vials showed different degrees of color change. The closer to the food surface, the less pronounced is the color change. Correspondingly, the lower half of the strip buried deeper into the food crack (1) has barely changed color.

Supplementary Table 1: Hydrogen peroxide treatment conditions and insect survival numbers: listed are the individual experiments per species (target H_2O_2 concentration and exposure time), the time points of insect survival assessment during the treatment time (TT) and post-treatment monitoring (PTM), and the survival numbers of experimental cohorts and control cohorts throughout the experiments; the size of each experimental and control cohort is given by the 0 h-value; V, 175 ml *Drosophila* vial; cage, 20 x 20 x 20 cm cage with netting; C, control; nr, not recorded.

Ae. aegypti

Experiment	TT (+ PTM)	Survival numbers experimental cohorts				Survival numbers control cohorts		
400 ppm, 7 h		V1	V2	V1 + V2		C1	C2	C3
	0 h	25	31			29	35	31
	7 h	13	16			29	35	31
	7 h (+ 14 h)	6	10	16		29	35	31
	7 h (+ 17 h)			15		29	35	31
	7 h (+ 40 h)			3		29	35	29
	7 h (+ 48 h)			0		29	34	28
400 ppm, 14 h		V4	V5			C1	C2	C3
	0 h	33	30			29	35	31
	14 h	0	0			29	35	31
400 ppm, 21 h		V3				C1	C2	C3
	0 h	44				29	35	31
	21 h	0				29	35	31
400 ppm, time to last death		V6	V7	V8	Cage 1	C1	C2	c3
	0 h	25	33	30	91	29	35	29
	7 h	18	24	16	57	nr	nr	nr
	8 h	8	14	10	23	29	34	28
	9 h	5	5	4	15	nr	nr	nr
	10 h	3	4	4	13	nr	nr	nr
	12 h	0	0	0	0	28	34	27
1000 ppm, 3 h		Cage 2				C1	C2	C3
	0 h	77				29	35	31
	3 h	0				29	35	31

An. stephensi

Experiment	TT (+ PTM)	Survival numbers experimental cohorts				Survival numbers control cohorts		
400 ppm, 7 h		V1	V2			C1	C2	C3
	0 h	40	31			34	28	36
	7 h	33	27			34	28	36
	7 h + 14 h	10	20			34	28	36
	7 h + 17 h	7	19			nr	nr	nr
	7 h + 40 h	3	5			33	27	36
	7 h + 48 h	1	3			33	27	36
400 ppm, 14		V4	V5	V4 + V5		C1	C2	C3
	0 h	35	37			34	28	36
	14 h	8	10	18		34	28	36
	14 h + 24 h			3		33	27	36
	14 h + 32 h			2		33	27	36
	14 h + 48 h			0		33	26	36
400 ppm, 21 h		V3				C1	C2	C3
	0 h	36				34	28	36
	14 h	nr				34	28	36
	21 h	0				34	28	36
400 ppm, time to last death		V6	V7	V8	Cage 1	C1	C2	C3
	0 h	33	34	40	65	33	28	36
	14 h	3	5	1	1	33	27	36
	15 h	2	5	1	0	nr	nr	nr
	16 h	0	5	1		nr	nr	nr
	17 h		5	0		nr	nr	nr
	18 h		1			nr	nr	nr
	19 h		1			nr	nr	nr
	20 h		1			nr	nr	nr
	21 h		0			33	27	36
1000 ppm, time to last death		Cage 2				C1	C2	C3
	0 h	46				34	28	36
	3 h	6				nr	nr	nr
	4 h	5				nr	nr	nr
	5 h	1				33	28	36

D. melanogaster

		**V1**	**V2**	**V3**	**V4**	**V5**	**C†**	**C2†**	**C3†**	
**400 ppm, 47 h, with food**	**0 h**	31	28	31	29	34	30	33	33	
	**7 h**	31	28	31	nr	nr	30	33	33	
	**14 h**	nr	nr	nr	29	34	30	33	33	
	**21 hr**	31	28	31	29	34	30	33	33	
	**28 h**	nr	nr	nr	29	34	30	33	33	
	**35 h**	31	28	31	27	34	30	33	33	
	**40 h**	nr	nr	nr	27	33	29	33	32	
	**42 h**	29	26	29	nr	nr	29	33	32	
	**47 h**	29	26	25	nr	nr	29	33	32	
	**40 h + 4.5 d**	nr	nr	nr	23	30	29	33	32	
	**47 h + 4.5 d**	21	15	15	nr	nr	29	32	32	
		**V6**	**V7**				**C1†**	**C2†**	**C3†**	
**1000 ppm, 5 h, with food**	**0 h**	33	24				30	33	33	
	**5 h**	33	24				30	33	33	
	**5 h + 15.5 h**	33	24				30	33	33	
	**5 h +24 h**	33	23				29	33	32	
	**5 h + 40 h**	nr	nr				29	33	32	
	**5 h + 2 d**	33	23				29	33	32	
	**5 h + 6 d**	32	23				29	32	32	
		**V8**	**V9**				**C4***	**C5***	**C6****	**C7****
**400 ppm, time to last death, no food**	**0 h**	35	34				58	51	64	45
	**6 h**	16	20				58	51	64	45
	**9 h**	0	0				54	47	60	45

† 20-25°C, 40-70% RH, with food; * 28°C, 40% RH, without food; ** 27°C, 70% RH, without food

C. capitata

		**Cage 1**	**Cage 2**	**C1**	**C2**	**C3**
**400 ppm, 26 h**	**0 h**	70	70	32	35	34
	**14 h**	63	60	32	33	34
	**17 h**	63	59	32	33	34
	**21 h**	57	51	32	33	34
	**26 h**	28	23	32	33	34
	**26 h + 4.5 d**	0	0	32	32	34
		**Cage 3**		**C1**	**C2**	**C3**
**1000 ppm, 5 h**	**0 h**	64		32	35	34
	**3 h**	64		32	35	34
	**4 h**	64		32	35	34
	**5 h**	59		32	35	34
	**5 h + 15.5 h**	51		32	33	34
	**5 h + 24 h**	47		32	33	34
	**5 h +48 h**	37		32	32	34
	**5 h + 6 d**	9		32	32	34
	**5 + 7d**	8		nr	nr	nr
	**5 + 8d**	6		nr	nr	nr
	**5 + 9d**	5		nr	nr	nr
	**5 + 10d**	4		nr	nr	nr

Sp. pandurus

		**Cage 1**	**Cage 2**	**C1**	**C2**	**C3**
**400 ppm, 47 h**	**0 h**	27	27	26	25	26
	**7 h**	27	27	26	25	26
	**21 h**	25	25	26	24	26
	**35 h**	25	25	26	24	26
	**42 h**	24	25	26	24	26
	**47 h**	21	25	26	23	26
	**47 h + 5 d**	19	15	23	23	19
	**47 h + 7 d**	6	12	22	21	18
		**Cage 3**		**C1**	**C2**	**C3**
**1000 ppm, 5 h**	**0 h**	22		26	24	26
	**3 h**	21		26	24	26
	**4 h**	21		26	24	26
	**5 h**	20		26	24	26
	**5 h +15.5 h**	20		26	24	26
	**5 h + 24 h**	20		26	24	26
	**5 h +48 h**	20		26	23	26
	**5 h + 6 d**	20		23	23	19
	**5 h + 8 d**	15		22	21	18

T. castaneum

**400 ppm, 47 h**		**V1**	**V2**	**V3**	**C1**	**C2**	**C3**
	0 h	33	33	34	32	33	34
	7 h	33	33	34	32	33	34
	21 h	33	33	34	32	33	34
	35 h	33	33	34	32	33	34
	42 h	33	33	34	32	33	34
	47 h	33	33	34	32	33	34
	47 h + 1 d	33	33	34	32	33	34
	47 h + 5 d	33	33	34	32	33	34
**1000 ppm, 5 h**		**V4**	**V5**		**C1**	**C2**	**C3**
	0 h	31	35		32	33	34
	3 h	31	35		32	33	34
	4 h	31	35		32	33	34
	5 h	31	35		32	33	34
	5 h + 15.5 h	31	35		32	33	34
	5 h + 1 d	31	35		32	33	34
	5 h + 2 d	31	35		32	33	34
	5 h + 6 d	31	35		32	33	34

Supplementary Table 2: Survival numbers of *Ae. aegypti* females upon heat exposure. Shown is the incubation temperature and duration, and the survival numbers of "young" and "old" *Ae. aegypti* females. Knock-down of females was recorded immediately after exposure termination and then in regular intervals for 24 h post exposure to account for temporary knock-down due to the heat. Tables report the highest number of alive females observed during the 24 h post treatment monitoring (PTM) period. The survival numbers recorded in the control cohorts („C“, 25°C) at the corresponding time points are shown in dark grey. For exposures conducted in parallel, only one control cohort was used. Experimental and control cohorts always consisted of 20 individuals. The color codes indicate the age span of each experimental cohort.

50°C

inc. time	young
2 min	19
2 min	15
2 min	17
5 min	0
5 min	0
5 min	0
C	20
C	20
C	20
C	20

2-4 days
2-7 days

48°C

inc. time	young	old
2 min	11	20
2 min	20	20
2 min	20	
5 min	0	1
5 min	0	0
5 min	7	
5 min	0	
8 min	0	
8 min	0	
8 min	0	
C	20	20
C	20	20
C	20	
C	20	

2-7 days | 15-17 days

45°C

inc. time	young
5 min	20
5 min	20
5 min	20
6 min	19
6 min	20
6 min	20
8 min	7
8 min	19
8 min	20
10 min	1
10 min	20
10 min	20
15 min	3
15 min	3
15 min	2
20 min	0
20 min	0
20 min	0
C	20
C	20
C	20
C	20
C	20

1-7 days
4-9 days

40°C

inc. time	young	old
15 min	20	20
15 min	20	20
60 min	20	15
60 min	20	20
60 min	20	
90 min	20	14
90 min	20	17
90 min	20	
2 h	18	9
2 h	17	0
2 h	20	18
2.5 h	19	1
2.5 h	19	9
2.5 h	20	
3 h	17	0
3 h	17	8
3 h	16	
4 h	11	8
4 h	7	2
4 h	9	
6 h	2	0
6 h	4	0
6 h	1	
6 h	2	
8 h	2	0
8 h	0	0
8 h	0	
9 h	0	
9 h	0	
9 h	0	
C	20	20
C	20	20
C	20	20
C	20	20
C	20	20
C	20	
C	20	

1-4 days | 10-15 days
2-7 days | 16-22 days
4-10 days

42°C

inc. time	young	old
10 min	20	20
10 min	20	20
20 min	19	19
20 min	20	18
20 min	20	
20 min	20	
30 min	20	20
30 min	20	9
30 min		20
30 min		3
40 min	14	4
40 min	20	0
40 min	9	12
40 min		14
50 min	19	1
50 min	17	0
50 min	18	1
50 min		6
60 min	9	12
60 min	13	16
60 min	15	0
70 min	10	3
70 min	6	0
70 min	8	0
80 min	7	5
80 min	3	0
80 min	6	
90 min	2	1
90 min	0	0
90 min	3	
100 min	0	0
100 min	0	0
100 min	0	
120 min	0	0
120 min		0
C	20	20
C	20	20
C	20	20
C	20	20
C	20	
C	20	
C	20	
C	20	

2-4 days | 10-14 days
2-7 days | 15-17 days
16-23 days
25-29 days

Supplementary Table 3. Survival numbers of heat treatments. Shown is the number of alive insects after incubation at the specified temperature and time. Each experimental and control cohort consisted of 20 individuals of mixed sex. In case of *Ae. aegypti* only the more temperature-resilient females were used and only young individuals (1-10 days after emergence) are shown here. A comparison of young and old *Ae. aegypti* females is shown in Suppl. Table 2 and Figure 8.

***Ae. aegypti* (females)**

	50°C	48°C	45°C	42°C	40°C
**2 min**	17	11			
**2 min**	19	20			
**2 min**	15	20			
**5 min**	0	0	20		
**5 min**	0	0	20		
**5 min**	0	7	20		
**5 min**		0			
**6 min**			19		
**6 min**			20		
**6 min**			20		
**8 min**		0	7		
**8 min**		0	19		
**8 min**		0	20		
**10 min**			1	20	
**10 min**			20	20	
**10 min**			20		
**15 min**			3		20
**15 min**			3		20
**15 min**			2		
**20 min**			0	19	
**20 min**			0	20	
**20 min**			0	20	
**20 min**				20	
**30 min**				20	
**30 min**				20	
**40 min**				14	
**40 min**				20	
**40 min**				9	
**50 min**				19	
**50 min**				17	
**50 min**				18	
**60 min**				9	20
**60 min**				13	20
**60 min**				15	20
**70 min**				10	
**70 min**				6	
**70 min**				8	
**80 min**				7	
**80 min**				3	
**80 min**				6	
**90 min**				2	20
**90 min**				0	20
**90 min**				3	20
**100 min**				0	
**100 min**				0	
**100 min**				0	
**2 h**				0	18
**2 h**					17
**2 h**					20
**2.5 h**					19
**2.5 h**					19
**2.5 h**					20
**3 h**					17
**3 h**					17
**3 h**					16
**4 h**					11
**4 h**					7
**4 h**					9
**6 h**					2
**6 h**					4
**6 h**					2
**6 h**					1
**8 h**					2
**8 h**					0
**8 h**					0
**9 h**					0
**9 h**					0
**9 h**					0
**C**	20	20	20	20	20
**C**	20	20	20	20	20
**C**	20	20	20	20	20
**C**	20	20	20	20	20
**C**	20	20	20	20	20
**C**				20	20
**C**				20	20
**C**				20	20
**C**				20	20
**C**				20	

D. melanogaster

	50°C	48°C	45°C	42°C	40°C
**2 min**	0	1	20		
**2 min**	0	1	20		
**2 min**	0	0	20		
**5 min**	0	0	0	20	
**5 min**	0	0	2	20	
**5 min**		0	0	20	
**7 min**				14	
**7 min**				20	
**7 min**				15	
**8 min**				17	
**8 min**				13	
**8 min**				16	
**10 min**			0	12	20
**10 min**			0	3	20
**10 min**				5	20
**15 min**				1	15
**15 min**				2	20
**15 min**				0	18
**20 min**					14
**20 min**					1
**20 min**				0	6
**20 min**				0	7
**25 min**				0	6
**25 min**				0	4
**25 min**					11
**25 min**					2
**30 min**					0
**30 min**					0
**30 min**					2
**60 min**					0
**60 min**					0
**60 min**					0
**C**	20	20	20	20	20
**C**	20	20	20	20	20
**C**	20	20	20	20	20
**C**	20	20	20	20	20
**C**			20	20	20
**C**			20	20	20
**C**				20	20
**C**					20
**C**					20

T. castaneum

	50°C	48°C	45°C	42°C	37°C
**2 min**	19	20			
**5 min**	12	19			
**10 min**	0	20	20		
**15 min**		17			
**20 min**		17	20		
**25 min**		3			
**30 min**		1	20		
**35 min**		0			
**40 min**			20		
**45 min**					
**50 min**			20		
**55 min**					
**60 min**			20	20	20
**1,5 h**			20	20	
**2 h**			20	20	
**3 h**			14	20	
**4 h**			5	20	
**5 h**			3	20	
**6 h**			0	20	
**8 h**				20	20
**24 h**				20	20
**32 h**				20	20
**48 h**				0	4
**72 h**					0
**C**	20	20	20	20	20
**C**	20	20	20	20	20
**C**	20	20	20	20	20
**C**		20	20	20	20
**C**		20	20	20	20
**C**		20	20	20	20
**C**		20	20	20	20
**C**		20	20	20	20

C. capitata

	50°C	48°C	45°C	42°C	40°C
**2 min**	20	20	20		
**2 min**	19	20	20		
**3 min**			15		
**3 min**			20		
**3 min**			20		
**5 min**	0	0	6		
**5 min**	0	0	18		
**5 min**			20		
**5 min**			20		
**6 min**			17		
**6 min**			15		
**6 min**			19		
**6 min**			19		
**7 min**			0		
**7 min**			17		
**7 min**			12		
**10 min**			0	20	
**10 min**			0	20	
**10 min**			3	20	
**15 min**			0	19	
**15 min**			0	9	
**15 min**				18	
**15 min**				18	
**20 min**				18	20
**20 min**				19	20
**20 min**				17	
**30 min**				9	20
**30 min**				4	19
**30 min**				9	20
**45 min**				3	15
**45 min**				6	16
**45 min**				9	19
**1 h**				0	13
**1 h**				0	11
**1 h**				3	13
**1.25 h**				0	
**1.25 h**				0	
**1.25 h**				0	
**1.5 h**					6
**1.5 h**					7
**1.5 h**					16
**2 h**					2
**2 h**					3
**3 h**					1
**3 h**					0
**3 h**					1
**4 h**					0
**4 h**					0
**C**	20	20	20	20	20
**C**	20	20	20	20	20
**C**	20	20	20	20	20
**C**			20	20	20
**C**			20	20	20
**C**			20	20	20
**C**			17	20	20
**C**			17	20	20
**C**				19	20
**C**					20
**C**					19
**C**					18
